# Impact of GDP, Spending on R&D, Number of Universities and Scientific Journals on Research Publications among Asian Countries

**DOI:** 10.1371/journal.pone.0066449

**Published:** 2013-06-20

**Authors:** Sultan Ayoub Meo, Abeer A. Al Masri, Adnan Mahmood Usmani, Almas Naeem Memon, Syed Ziauddin Zaidi

**Affiliations:** 1 Department of Physiology, University Diabetes Centre, College of Medicine, King Saud University, Riyadh, Saudi Arabia; 2 University Diabetes Centre, College of Medicine, King Saud University, Riyadh, Saudi Arabia; 3 Department of Radiology, Isra University, Hyderabad, Pakistan; 4 Department of Hematology and Bone Marrow Transplantation, King Fahad Medical City, Riyadh, Saudi Arabia; University of Warwick, United Kingdom

## Abstract

**Objectives:**

This study aimed to compare the impact of Gross Domestic Product (GDP) per capita, spending on Research and Development (R&D), number of universities, and Indexed Scientific Journals on total number of research documents (papers), citations per document and Hirsch index (H-index) in various science and social science subjects among Asian countries.

**Materials and Methods:**

In this study, 40 Asian countries were included. The information regarding Asian countries, their GDP per capita, spending on R&D, total number of universities and indexed scientific journals were collected. We recorded the bibliometric indicators, including total number of research documents, citations per document and H-index in various science and social sciences subjects during the period 1996–2011. The main sources for information were World Bank, SCI-mago/Scopus and Web of Science; Thomson Reuters.

**Results:**

The mean per capita GDP for all the Asian countries is 14448.31±2854.40 US$, yearly per capita spending on R&D 0.64±0.16 US$, number of universities 72.37±18.32 and mean number of ISI indexed journal per country is 17.97±7.35. The mean of research documents published in various science and social science subjects among all the Asian countries during the period 1996–2011 is 158086.92±69204.09; citations per document 8.67±0.48; and H-index 122.8±19.21. Spending on R&D, number of universities and indexed journals have a positive correlation with number of published documents, citations per document and H-index in various science and social science subjects. However, there was no association between the per capita GDP and research outcomes.

**Conclusion:**

The Asian countries who spend more on R&D have a large number of universities and scientific indexed journals produced more in research outcomes including total number of research publication, citations per documents and H-index in various science and social science subjects.

## Introduction

Research in science and social science sectors play an important role in the country’s communal and economic growth along with long-term sustainable development. The research innovations in science and social sciences contribute to improving the living standards and quality of life. Considering the rising significance of research in economic growth of a nation, many countries are reducing dependency on their natural resources and swiftly moving towards knowledge-based economy. Investment in research is important for the progress in science and technology as well as for social and economic development. [1].

To recognize and quantify the progress of research, bibliometric indicators are essential tools to understand the size, growth and global spread of research. Bibliometric indicators are frequently practiced to measure the scientific productivity, visibility and capacity of research publications with global science. These indicators are mainly based on the number of scientific research documents (research papers) published and global citations received (cited by other researchers). Bibliometric indicators quantify the quantity, quality of research output and there are structural indicators as well which assess the association between authors, publications and areas of research in universal science [2].

Science and technology cannot exist if researchers do not evidence or publish their experimental findings and results. Scientific writing and its outcome in the form of research publication are essential components of academic excellence. Scientific publications are a key indicator of the development of a country, and a healthy scientific research environment is a prerequisite for scientific and economic progress [3].

The research information seeking behavior is essential in economic success of a country [4]. In order to achieve long-term and sustainable economic growth, spending on education, research and development is essential to produce a substantial amount of innovative research. There is a direct relationship between research and the overall development of nations. Keeping all these facts in mind, the present study aimed to compare the impact of Gross Domestic Product (GDP), spending on Research and Development (R&D), number of universities and indexed scientific journals in Institute of Scientific Information (ISI) on bibliometric indicators including total number of research documents (research papers), citations per document (referenced/cited in the literature), and Hirsch index (H-index) an index to quantify an individual’s scientific research output, in various science and social science subjects among Asian countries.

## Materials and Methods

This observational study was conducted in the Department of Physiology, College of Medicine, King Saud University, Riyadh, Saudi Arabia. In this study, we reviewed all the 50 Asian countries [5]. The countries who published less than 100 publications per annum during the period 1996–2011 were excluded from the study. Finally, for this study, we included 40 Asian countries. The information regarding all the Asian countries, their average per capita GDP and total GDP for the last five years, spending on R&D were collected from the World Bank sources [6], and the data about number of universities were collected from the World Association of Universities [7]. The information regarding scientific journals which are indexed in Institute of Scientific Information (ISI) was obtained from Web of Science, Institute of Scientific Information (ISI) Journal Citation Reports (Thomson Reuters) [8]. Bibliometric indicators in Science and Social Science subjects during the period 1996–2011 were recorded from SCI-mago/Scopus [9]. For ISI indexed journals, we logged on to Web of Science, the territory was selected, country name was entered, and the names of journals along with impact factors for each Asian country were retrieved. For the recording of bibliometric indicators, research outcome in all world scientific journals indexed in Scopus was recorded. In SCI-mago site, region and country was selected, subject field “Science” and “Social Sciences” were selected and detailed information regarding the bibliometric indicators including total number of research papers (documents), citations per document and H-index in science and social science subjects among Asian countries were obtained.

### Statistical Analysis

The data were analyzed by using Statistical Package for the Social Sciences (SPSS) software version 18. Data were expressed as Mean ± Standard Error of Mean (SEM). The Pearson correlation coefficient and Kendall’s rank correlation coefficient were calculated to find the strength of relation between different variables. p-value <0.05 was considered significant.

## Results

The total numbers of Asian countries included in this study are 40. The mean per capita GDP for all the Asian countries is 14448.31±2854.40 US$. The yearly per capita spending on R&D is 0.64±0.16 US$, number of universities 72.37±18.32 and mean number of ISI indexed journal per country is 17.97±7.35 (Table 1).

**Table 1:** Asian countries with their GDP per capita, spending on R&D as% of total GDP, number of universities and scientific indexed journals.

Countries	GDP in US$	Spending on R&D (% of total GDP)	Universities	Journals
Armenia	3184.33	0.220	12	3
Azerbaijan	5427.03	0.193	26	1
Bahrain	18867.55	0.2	11	2
Bangladesh	607.96	0.080	80	4
Brunei Darussalam	32063.81	0.040	3	0
Cambodia	764.05	0.100	20	0
China	3938.31	1.418	376	155
Cyprus	29733.22	0.438	15	0
Georgia	2698.83	0.213	13	0
Hong Kong	31372.38	0.775	12	0
India	1214.78	0.762	321	100
Indonesia	2550.01	0.083	174	0
Iran	4402.87	0.728	186	39
Iraq	2582.35	0.100	35	0
Israel	27340.55	4.544	21	13
Japan	40101.26	3.431	567	236
Jordan	3976.35	0.421	29	1
Kazakhstan	8552.92	0.224	24	0
Kuwait	50566.56	0.091	6	3
Lebanon	8146.10	0.300	24	0
Macao	46672.87	0.086	3	0
Malaysia	7987.01	0.635	146	10
Mongolia	2143.60	0.230	10	0
Nepal	477.89	0.100	7	1
Oman	20524.01	0.190	11	0
Pakistan	1002.35	0.569	128	13
Palestine	1194.33	0.100	15	0
Philippines	1991.05	0.110	113	6
Qatar	75175.82	2.000	2	0
Saudi Arabia	16861.75	0.058	61	6
Singapore	38934.97	2.401	6	50
South Korea	20108.20	3.193	142	0
Sri Lanka	2184.17	0.144	24	1
Syria	2590.35	0.200	15	0
Thailand	4211.33	0.231	65	8
Turkey	9729.07	0.719	86	54
United Arab Emirates	44544.27	0.150	31	12
Uzbekistan	1191.56	0.120	22	1
Viet Nam	1135.71	0.120	41	0
Yemen	1181.09	0.110	12	0
**Mean**	**14448.31**	**0.64**	**72.37**	**17.97**
**SEM**	**2854.40**	**0.16**	**18.32**	**7.35**

Main sources of retrieving information [6]–[8], Data expressed as Mean±SEM.

Table 2 shows the number of total documents published in various science and social sciences subjects among the Asian countries during the period 1996–2011 as 158086.92±69204.09; citations per document 8.68±0.48; and H-index 122.8±19.21.

**Table 2:** Asian countries with total number of research documents, citation per documents and H-Index.

Countries	Research Documents	Citations/document	H-index
Armenia	8054	8	98
Azerbaijan	6135	3	41
Bahrain	2817	5	36
Bangladesh	16074	8	89
Brunei Darussalam	1064	10	37
Cambodia	1296	12	45
China	2248278	6	353
Cyprus	8427	12	79
Georgia	6381	8	71
Hong Kong	144935	14	268
India	634472	8	281
Indonesia	16139	11	103
Iran	159046	8	121
Iraq	4420	5	37
Israel	204262	17	393
Japan	1604017	12	602
Jordan	17126	7	72
Kazakhstan	4695	4	46
Kuwait	12254	7	77
Lebanon	11672	9	91
Macao	1559	6	31
Malaysia	75530	8	116
Mongolia	1723	13	51
Nepal	5092	9	66
Oman	6875	7	58
Pakistan	47443	6	101
Palestine	2273	8	41
Philippines	11326	13	107
Qatar	4398	6	44
Saudi Arabia	46167	7	114
Singapore	126881	13	240
South Korea	497681	10	309
Sri Lanka	7019	10	78
Syria	3379	10	53
Thailand	69637	11	156
Turkey	267902	8	193
United Arab Emirates	15698	7	81
Uzbekistan	6763	4	50
Viet Nam	13172	12	101
Yemen	1395	7	34
**Mean**	**158086.92**	**8.68**	**122.8**
**SEM**	**69204.09**	**0.48**	**19.21**

Main source of retrieving information [9], Data expressed as Mean±SEM.

Table 3 demonstrates the Pearson correlation coefficient between the mean GDP per capita, spending on R&D, number of universities, indexed journals and total number of research documents, citations per document, H-index in various science and social sciences subjects among Asian countries during the period 1996–2011.

**Table 3:** Pearson correlation coefficient between GDP per capita, spending on R&D as% of total GDP, number of universities, indexed journals and total number of research documents, citations per document, H-index in various science and social sciences subjects among Asian countries during the period 1996–2011.

Parameters	Research Documents	Citation per documents	H-Index
**GDP per capita US$**	r = 0.050	r = 0.064	r = 0.189
	p = 0.761	p = 0.694	p = 0.242
**Spending on R&D**	r = 0.480	r = 0.429	r = 0.805
	p = 0.002	p = 0.006	p = 0.0001
**Universities**	r = 0.841	r = 0.063	r = 0.755
	p = 0.0001	p = 0.699	p = 0.0001
**Indexed journals**	r = 0.893	r = 0.085	r = 0.801
	p = 0.0001	p = 0.602	p = 0.0001

r = Pearson correlation coefficient.

p = p-value.

**Figure 1:**
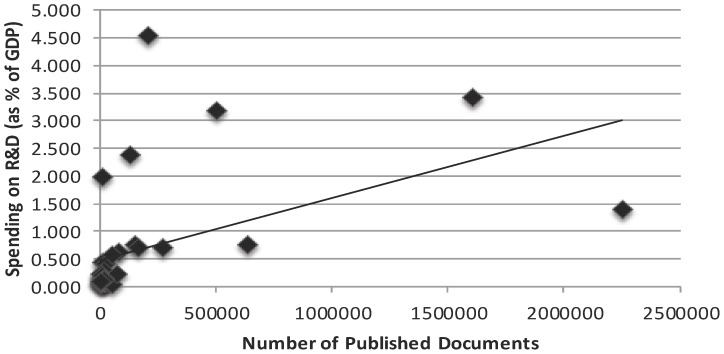
Correlation coefficient between spending on R&D and total number of research documents in various science and social science subjects among Asian countries during the period 1996–2011.

**Figure 2:**
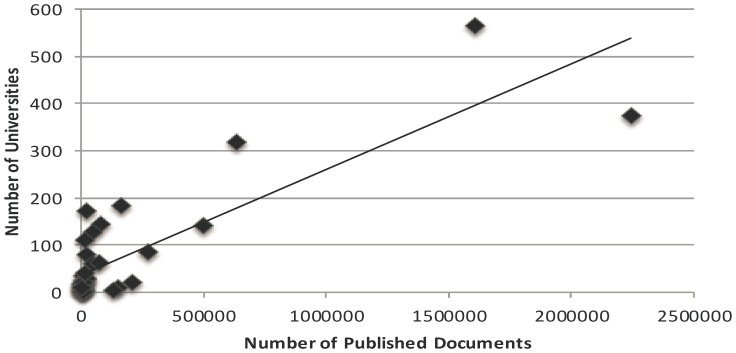
Correlation coefficient between the number of universities and total number of research documents in various science and social science subjects among Asian countries during the period 1996–2011.

**Figure 3:**
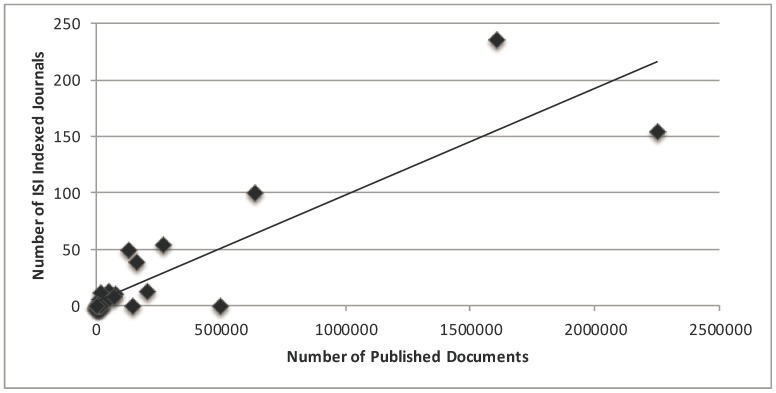
Correlation coefficient between scientific indexed journals and total number of research documents in various science and social science subjects among Asian countries during the period 1996–2011.

**Figure 4:**
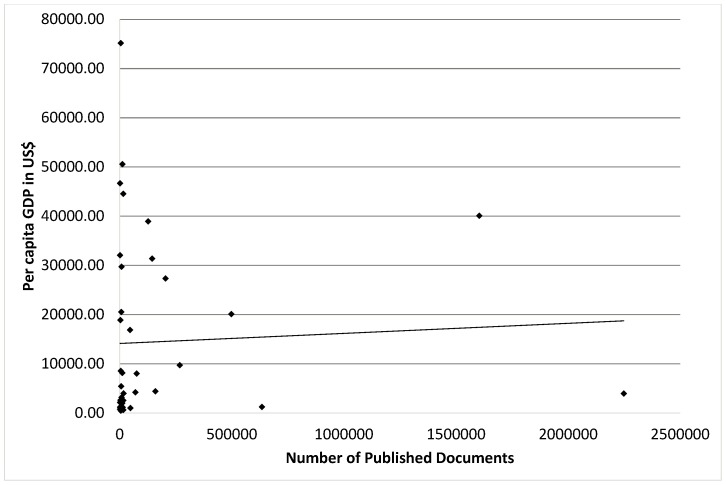
Correlation coefficient between per capita GDP and total number of research documents in various science and social science subjects among Asian countries during the period 1996–2011.

We found that spending on R&D has a positive correlation with total number of published documents (r = 0.480; P = 0.002) [Fig. 1], citations per documents (r = 0.429; P = 0.006) and H-Index (r = 0.805; P = 0.0001) [Table 3]. In addition, we found that number of universities has a positive correlation with total number of documents (r = 0.841; P = 0.0001) [Fig. 2], and H-Index (r = 0.755; P = 0.0001) [Table 3]. Moreover, we also found that ISI indexed journals have a positive correlation with total number of published documents (r = 0.893; P = 0.0001) [Fig. 3], and H-Index (r = 0.801; P = 0.0001) [Table 3]. However, we did not find strong positive correlation between per capita GDP and research outcomes [Table 3, Fig. 4].

In addition to Pearson correlation coefficient, we also applied the Kendall’s tau (τ) rank correlation coefficient between different variables. We found that spending on R&D has a positive Kendall’s tau (τ) rank correlation coefficient with total number of published documents (τ = 0.447; P = 0.0001) and H-Index (τ = 0.457; P = 0.0001). In addition, we found that number of universities has a positive correlation with total number of documents (τ = 0.532; P = 0.0001) and H-Index (τ = 0.475; P = 0.0001). Furthermore, we found that ISI indexed journals have a positive correlation with total number of documents (τ = 0.601; P = 0.0001) and H-Index (τ = 0.536; P = 0.0001) [Table 4].

**Table 4:** Kendall’s rank correlation coefficient between GDP per capita, spending on R&D, number of universities, indexed journals and total number of research documents, citations per document, H-index in various science and social sciences subjects among Asian countries during the period 1996–2011.

Parameters	Documents	Citation per documents	H-Index
**GDP per capita (US$)**	τ = 0.136	τ = −0.032	τ = 0.107
	p = 0.217	p = 0.778	p = 0.333
**Spending on R&D**	τ = 0.447	τ = 0.139	τ = 0.457
	p<0.0001	p = 0.226	p<0.0001
**Universities**	τ = 0.532	τ = 0.027	τ = 0.475
	p<0.0001	p = 0.814	p<0.0001
**Indexed journals**	τ = 0.601	τ = 0−0.02	τ = 0.536
	p<0.0001	p = 0.872	p<0.0001

τ (tau) = Kendall’s rank correlation coefficient.

p =  p value.

## Discussion

The role of research in driving the productivity and pecuniary growth is important in which knowledge is central to economic development. Few Asian countries experienced a shift from their natural resources, agriculture, and primary commodities dependent economy to knowledge-based economy [10]–[11].

In this study, we compared the impact of GDP per capita, spending on R&D, number of universities and scientific indexed journals on bibliometric indicators including total number of research documents, citations per document and H-index in various science and social sciences subjects during the period 1996–2011. We found a positive correlation between the spending on R&D, number of universities, indexed journals and total number of research documents, citations per document and H-Index in various science and social science subjects in Asian countries. However, we did not find an association between per capita GDP and research outcomes (Table 3–4).

**Figure 5:**
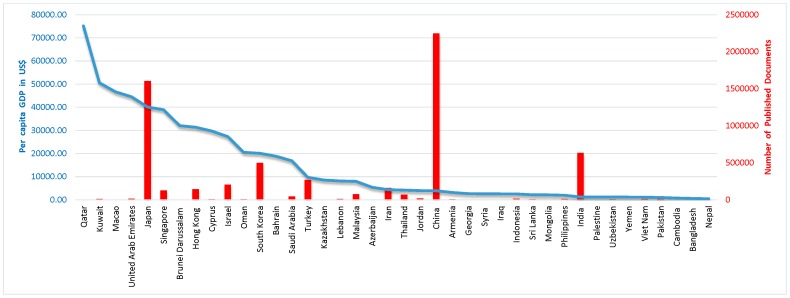
Association between per capita GDP and total number of research documents published in various science and social sciences subjects among Asian countries during the period 1996–2011.

GDP is the economic growth measured in terms of an increase in the size of country economy. It is the main indicator used to gauge the strength of a country’s economy and represents the total value of all goods and services produced over a specific time period. We found no correlation between per capita GDP and total number of documents, citations per document and H-Index in various science and social science subjects (Table 3–4, Fig. 5). The Asian countries with the highest GDP are Qatar, Kuwait, Macao, and United Arab Emirates and the countries with the lowest GDP are Cambodia, Bangladesh and Nepal (Table 1). It is well-known that Qatar, Kuwait, Macao, and United Arab Emirates are among the richest in Asia, but their research publications are in contrast to their GDP (Fig. 5). In Japan, South Korea and Turkey research growth is comparable to their GDP. Although GDP of China and India is low, yet they are producing high in research outcomes (Fig. 5). In the present study, we found that there is no difference in the research outcomes between the countries with high GDP compared to the countries with a low GDP. The results of the present study show that, the research outcome does not depend upon GDP, but it actually depends on how much percentage of total GDP is being spent on R&D. The investment in R&D is a major factor in determining the contribution that research can make to scientific progress and innovation. The investment in R&D is associated with high rates of research outcomes which lead to knowledge based economy.

The national innovative capacity is highly dependent on R&D investment [12]–[13]. The Asian countries spending more on R&D are Israel, Japan, South Korea, Singapore, Qatar and to some extent, China, Saudi Arabia, Hong Kong, India, Iran and Turkey. These countries are spending more on R&D and generating more in research outcomes. The annual spending on R&D in Asian countries especially the wealthy Arab states is just 0.2% of the gross national product compared to the world average of 1.4% [14]–[15]. It has also been reported that average annual spending on R&D by most of the Organization of Islamic Countries (OIC) in Asia was 0.34% of GDP, much lower than the global average over the same period as 2.3% [15]. However, in recent years, few Arab states in Asia including Qatar, Saudi Arabia, Kuwait and UAE resolved to spend more on R&D. The average spending on R&D in the Asian countries is 0.64±0.16 (Table 1), it is still small, but it shows a positive correlation with research outcomes.

Zhang [16] conducted a study on the research relationship between expenditure for science and technology activities and economic growth in China and reported that R&D input adds great contribution in the economic growth development. The R&D efforts provide better opportunities to create new knowledge and enhance capabilities to integrate and exploit external knowledge. Therefore, investing greater amount on R&D generate strong research and technological based capabilities in terms of their processes and product innovations and enhances overall performance.

Helpenny et al. [17] conducted a study to examine the geographic origin of publications and link between the percentages of GDP spent on R&D. They found that the percentage of GDP spent on R&D was positively correlated with the number of publications (r = 0.603, P<.001). Similarly, in the present study, we found a positive correlation between the spending on R&D and research outcomes in various science and social science subjects. Our study findings are in agreement with the results of Helpenny et al. [17]. Anwar and Abu Baker [15] have reported that most of the Asian Arab world countries lack researchers, scientists and technicians, and have an average of nine scientists, engineers and technicians per thousand people, compared with a world average of forty-one. The obvious reason is meager spending on R? hence the majority of Asian countries have less number of researchers and scientists. Furthermore, it has been reported that from Asian countries, a large number of the research scholars, scientists migrate to other continents including North America, Europe and Australia. A more recent report published in Nature indicates that, 12% researchers, scientists from China, 37% from India have migrated to UK, USA and Australia [18]. Moreover, majority of researchers and scientists from other Asian countries including Pakistan, Bangladesh and Jordan is also moving to cross-continent. The scientific travel is not only about empirical observation but the scientists also carry with them sort of scientific attestation. This large figure of researchers immigrating to other countries creates a gap. Besides this situation, we found a positive correlation between spending on R&D and research out comes (Table 3, Fig.1).

In Asian countries, the number of universities and research institutions is also not satisfactory enough, the mean number of universities per country in Asia is 72.37±18.32, and the mean population of these countries is 101334431.8±144636036.51. The number of universities is very small compared to the population of these countries. In spite of this situation, in the present study we found a positive correlation between the number of universities and H-Index in science and social science subjects among the Asian countries (Table 3, 4, Fig.2).

Choung and Hwang [19] reported that universities play an important role in increasing number of research papers in the ISI database and the related research activities of the universities supported the development of industrial technologies. Similarly, in the present study we found a positive association between number of universities and an increasing number of ISI research papers. This is an established fact that the basic place of the research is the universities, we believe, Asian countries must increase number of universities and eventually the research outcome will further enhance.

In addition to reviewing GDP, spending on R&D, number of universities, we also reviewed the ISI indexed journals in Asian countries. The countries in Asia having a large number of ISI indexed journals are as follows: Japan 236, China 155, India 100, Singapore 55, Turkey 54, and Iran 39 (Table 1). The mean number of journals in the all the Asian countries is 17.97±7.35 (Table 1) with an average impact factor 0.58. It is evident that the scientific journals; especially the ISI indexed journals, are less in number. Many do not have on-line access, and are not indexed in major bibliographic/citation databases. The majority of indexed journals, however, do not have a stable presence in popular scientific databases. The Arab Asian countries are producing less than 0.5% of scientific research papers appearing in the 200 leading medical journals. Furthermore, the number of publications, original writings and translations per million people is about 0.05 in the Arab Asian countries, compared with an average of 0.15 worldwide and 0.6 in industrialized countries [20]. In the present study, we found that the total number of ISI indexed journal in Asian countries is 719 (Table 1). This shows that the total number of ISI indexed journals in Asian countries is even less than a single European country like UK that has 1637 ISI indexed journals [8]. We found a positive correlation between the number of scientific journals and total number of research documents and H-index; it shows that countries having more ISI Indexed journals are producing more research papers (Table 3, 4, Fig.3).

### Study Strengths and Limitations

The main strengths of this study are that; we selected the large number of Asian countries, employed all the promising parameters to compare the research outcomes which play potential role in the development of research such as GDP, spending on R&D, number of universities, and ISI-Indexed Journals. We collected the information regarding the Asian countries, their GDP, spending on R&D, from very reliable source of the World Bank. The data about number of universities were gained from the World Association of Universities. The information regarding the ISI-indexed scientific journals and Bibliometric indicators in various science and social sciences subjects were obtained from the Institute of Scientific Information (ISI), Web of Science, Journal Citation Reports (Thomson Reuters), and SCI-imago web. These are highly reliable sources in scientific literature. However, sometimes citation count tools may mis-cite a paper, and there are chances of same paper may appear twice with slightly different details. This may inflate the number of citation counts. This is one of the limitations of the present study.

### Conclusion

This is the first study which has analyzed the productivity and visibility of research papers in Asian countries. We found that spending on R&D, number of universities and scientific indexed journals have a positive association with the total number of research documents, citations per documents and H-index in various science and social science subjects. However, we did not find an association between the per capita GDP and research outcomes. It shows that the Asian countries who are spending more on R&D, have more universities and ISI indexed journals are producing significant volume of research papers. It is suggested that Asian countries need to recognize the importance of scientific research for social and economic development. They also need to establish more universities, increase funding for R&D and launch more scientific journals and must get indexed with ISI. Continuing efforts must be taken to develop the policy to promote research culture. These steps will augment the research oriented education and culture and ultimately the research outcome will increase and bring the scientific, social as well as economic development in the region.
