# Correction: Impact of GDP, Spending on R&D, Number of Universities and Scientific Journals on Research Publications among Asian Countries

**DOI:** 10.1371/annotation/3a739c2a-d5f2-4d6f-9e0d-890d5a54c33d

**Published:** 2013-10-28

**Authors:** Sultan Ayoub Meo, Abeer A. Al Masri, Adnan Mahmood Usmani, Almas Naeem Memon, Syed Ziauddin Zaidi

Funding information was incorrectly given in the Acknowledgements section.

In addition, a funding organization was incorrectly given in the Funding Statement. The Funding Statement should read: "The authors are thankful to the College of Medicine Research Centre (CMRC), Deanship of Scientific Research, King Saud University, Riyadh, Saudi Arabia, for supporting the work. The funders had no role in study design, data collection and analysis, decision to publish, or preparation of the manuscript." 

